# The Cancer Differential: Minorities in Racially Segregated Urban Areas at Higher Risk than Whites

**DOI:** 10.1289/ehp.114-a176b

**Published:** 2006-03

**Authors:** Tanya Tillett

Nearly 80% of the U.S. population lives in metropolitan areas. With continued growth of urban centers has come increased study of the connection between the built environment, social inequality, and the health and well-being of inhabitants of these large cities. A number of factors related to neighborhood location and other area-level variables such as access to nutritious foods and health care can affect human health. Now a research team points to another health consideration, demonstrating that minority populations living in highly segregated metropolitan areas in the United States have higher estimated lifetime cancer risks from air toxics than whites **[*EHP*** **114:386–393; Morello-Frosch et al.]**.

The team analyzed more than 45,000 census tracts in 309 U.S. metropolitan areas for level of segregation. The metropolitan areas were classified as low-to-moderately segregated, highly segregated, or extremely segregated, based on the proportion of people who would have to move to achieve an even racial balance in every neighborhood of the city. They also used census data to divide racial and ethnic groups into six categories: Hispanics of any race, non-Hispanic whites, non-Hispanic blacks, non-Hispanic Asians and Pacific Islanders, non-Hispanic American Indians and Alaska Natives, and non-Hispanic persons of other race.

Then the team used federal air toxics data for 1996 to derive cancer risk estimates. Cancer risks were determined using inhalation unit risk estimates for each known, likely, or potential human carcinogen measured in the tracts’ air. Inhalation unit risk estimates consider the individual lifetime excess risk resulting from chronic lifetime exposure to one unit of pollutant concentration.

The researchers found a persistent relationship between increasing levels of racial/ethnic segregation and increased estimated cancer risk associated with ambient air toxics. Hispanics in extremely segregated areas were the most affected, with a 6.4-fold increased lifetime cancer risk compared to Hispanics in low-to-moderately segregated areas. Non-Hispanic American Indians and Alaska Natives in highly segregated areas were the least affected, with a 1.39-fold increased risk over their counterparts in low-to-moderately segregated areas. The influence of racial segregation on cancer risk appeared independent of the effect of poverty across racial categories. The most significant contributors to cancer risk were mobile sources such as on-road vehicles, airplanes, and trains, with diesel emissions an overwhelming source of pollution.

The authors note that these results are consistent with findings from a previous national study that analyzed the relationship between black/white residential segregation and ambient air toxic exposure in U.S. metropolitan areas. They believe this study to be the first examination of environmental health disparities to use a generalized multiethnic segregation measure. They assert that future research on this issue that incorporates new and better models of exposure should include segregation as a key factor in analysis.

